# Porcine Model of Cerebral Ischemic Stroke Utilizing Intracortical Recordings for the Continuous Monitoring of the Ischemic Area

**DOI:** 10.3390/s24102967

**Published:** 2024-05-07

**Authors:** Thomas Gomes Nørgaard dos Santos Nielsen, Numa Dancause, Taha Al Muhammadee Janjua, Felipe Rettore Andreis, Benedict Kjærgaard, Winnie Jensen

**Affiliations:** 1 Bevica Center, Department of Health Science and Technology, Aalborg University, Selma Lagerløfs Vej 249, 9260 Gistrup, Denmark; 2 Département de Neurosciences, Université de Montréal, C.P. 6128 Succursale Centre-Ville, Montréal, QC H3C 3J7, Canada; 3 Center for Neuroplasticity and Pain (CNAP), Department of Health Science and Technology, Aalborg University, Selma Lagerløfs Vej 249, 9260 Gistrup, Denmark; 4 Department of Cardiothoracic Surgery, Aalborg University Hospital, Hobrovej 18, 9000 Aalborg, Denmark

**Keywords:** ischemic stroke, electrophysiology, endothelin-1, intracortical recordings, micro-electrode array, pig

## Abstract

Purpose: Our aim was to use intracortical recording to enable the tracking of ischemic infarct development over the first few critical hours of ischemia with a high time resolution in pigs. We employed electrophysiological measurements to obtain quick feedback on neural function, which might be useful for screening, e.g., for the optimal dosage and timing of agents prior to further pre-clinical evaluation. Methods: Micro-electrode arrays containing 16 (animal 1) or 32 electrodes (animal 2–7) were implanted in the primary somatosensory cortex of seven female pigs, and continuous electrical stimulation was applied at 0.2 Hz to a cuff electrode implanted on the ulnar nerve. Ischemic stroke was induced after 30 min of baseline recording by injection of endothelin-1 onto the cortex adjacent to the micro-electrode array. Evoked responses were extracted over a moving window of 180 s and averaged across channels as a measure of cortical excitability. Results: Across the animals, the cortical excitability was significantly reduced in all seven 30 min segments following endothelin-1 injection, as compared to the 30 min preceding this intervention. This difference was not explained by changes in the anesthesia, ventilation, end-tidal CO_2_, mean blood pressure, heart rate, blood oxygenation, or core temperature, which all remained stable throughout the experiment. Conclusions: The animal model may assist in maturing neuroprotective approaches by testing them in an accessible model of resemblance to human neural and cardiovascular physiology and body size. This would constitute an intermediate step for translating positive results from rodent studies into human application, by more efficiently enabling effective optimization prior to chronic pre-clinical studies in large animals.

## 1. Introduction

In 2019, 12.2 million people had a stroke worldwide, of which 62.4% (7.63 million) were ischemic. Despite the growing number of stroke incidents and stroke survivors, the age-adjusted incidence and prevalence of ischemic stroke were lower in 2019 than in 1990 at 94.51 (−10%) and 951.0 (−2%), respectively. As such, the age-adjusted stroke incidence and prevalence in people under 70 years have increased, and the age-adjusted stroke incidence declined less than in the preceding decade, while the prevalence simultaneously increased in high-income countries [1]. Improvements in ischemic stroke outcomes in high-income countries have been facilitated, e.g., by thrombolysis and thrombectomy treatments, using individualized treatment enabled by imaging technologies, improvement of hospital management and rescue services, and awareness campaigns for stroke symptoms have reduced the crucial time window between when the patient was last known to be well and reperfusion [1,2,3].

Neuroprotective strategies (i.e., methods that reduce detrimental processes in the brain through means other than re-establishing cerebral blood flow [4]) may be key to continued improvements in patient outcomes for the future because the potential for tissue survival is higher within the first hour [5]. The timing issue of a neuroprotective intervention may be particularly important for neuroprotective agents that mitigate the effects, e.g., of excitotoxicity, which would require early administration to be effective, but may also impact the benefit of, e.g., anti-inflammatory mechanisms because they may be beneficial and detrimental at different stages of infarct and recovery [6]. The underlying mechanisms of the hyperacute or acute progression of ischemia, and tools to study these, are therefore of importance.

Animal experiments are pivotal to stroke research because they allow for the study of both the mechanisms and treatments of stroke using invasive measurements and enabling interventions that model specific or general aspects of stroke. However, over the past few decades, multiple pharmaceuticals have been identified and have shown great promise in (mostly rodent) animal models, but successful implementation in clinical trials has remained elusive [4,7,8]. There are many potential explanations for the failure to translate encouraging results from animals to human applications, including the physiological differences between rodents and humans, the unrealistic lack of comorbidities in young animals, as well as the sub-optimal design of clinical trials, e.g., grouping the results of intervention windows of up to 72 h, which may blur positive results in sub-groups of patients [4,8,9]. Therefore, there has been a scientific focus on developing translational preclinical large animal models that can bridge the gap between animal studies and human clinical trials. Among these models, pigs have gained significant attention due to their similarities to humans in terms of proteomics, genomics, and immunology [10]. Also, pigs are more easily accessible than monkeys. Pigs also have a gyrencephalic brain with a similar grey-to-white matter ratio as humans, they are comparable in size to some non-human primates [11,12], and have a body weight and cardiovascular system similar to those of humans [12].

Several methods have been developed to mimic focal cerebral ischemia in animals, including mechanical occlusion, embolic occlusion, or vasoconstriction. Studies of ischemic stroke have also previously been performed in pigs using the photothrombotic model [13], mechanical MCA occlusion models [14,15], and intracortical injection of endothelin-1 (ET-1) [16,17,18,19]. The ET-1-based models are of particular interest because they are relatively simple experimentally and, depending on the method of application, can create very localized ischemia (like that of the photothrombotic model) or more physiologically realistic arterial restriction (like that of mechanical models) and allow for the study of reperfusion as the effects of the agent dissipate. ET-1 can be applied on the surface of the cortex or dura [20,21], injected into cortical structures [22,23,24], or through injection near the arterial wall of the MCA [25,26,27], providing a far less invasive means for MCA occlusion than mechanical models [15].

To monitor and assess the progression of an ischemic infarct and possible interventions in pigs, the most used methodologies are neuroimaging, histology, and/or behavioral assessment. Histology can provide insights on, e.g., the infarct volume and changes at the cellular level. Histological samples are gathered at the termination of an experiment and, therefore, can reveal the state of the brain at one fixed time point. Imaging techniques like MR, CT, or PET can be used to assess the cerebral blood flow (CBF) and cerebral blood volume (CBV) to estimate the infarct location and volume (typically not less than 10–15 min between measurements, see, e.g., [16,18]). The use of intracortical recordings is a well-established method to study the cell functionality, coding, and plasticity of the mammalian brain in animals [8,9]. The use of intracortical recordings allows us to study the intricate details of the brain response at a higher spatial resolution and temporal resolution than the more traditional assessment methods. Examples of the use of intracortical electrophysiological recordings in pigs are, however, sparse in the literature [28,29,30,31,32]. The use of somatosensory evoked potentials has been used for identifying the location and topographical organization of the somatosensory cortex [33,34,35,36] or for studying cortical mechanisms [28,34,37].

Pig models are believed to serve as a better translational model than rodent models and are easier to work with than non-human primates, as stated above. Pig models have previously been used to study stroke, but not utilizing cortical recordings to analyze the effect of ischemic stroke. The aim was therefore to use intracortical recordings to enable the tracking of infarct development in the penumbra over the first few critical hours of ischemia with a high time resolution. We induced a focal infarction by applying ET-1 on the cortex and used somatosensory evoked potentials to assess the cortical excitability.

## 2. Materials and Methods

All experimental procedures reported in this section were planned prior to the experiments but were not published, in part, because adjustments were expected to be necessary due to the exploratory nature of this study. During initial experiments, data were inspected after each experiment, mainly to evaluate data quality. Based on observations made during this offline inspection as well as during the experiments, the full data processing pipeline reported in this section was decided before performing the analysis. All methods were carried out in accordance with relevant guidelines and regulations and in accordance with the ARRIVE and 3R guidelines.

### 2.1. Surgical Procedures

#### 2.1.1. Animals and Anesthesia

All experimental procedures were approved by the Animal Experiments Inspectorate under the Ministry of Food, Agriculture and Fisheries of Denmark, approval no. 2020-15-0201-00401. Fifteen female pigs were included in this study (female pigs were included simply for practical reasons such as housing and ease of placement of catheters). The pigs were brought to the animal facility and acclimatized for at least two weeks prior to experimentation. In this period, they were housed in a room (∼24 °C, 13/11 h light/dark cycle) with at least one other pig (except for the last day for the last pig of a group) and had free access to food and water. The pigs (Danish Landrace), weighing 32.7 ± 3.1 kg (mean ± standard deviation) (approx. 12 weeks old), were initially sedated and anesthetized with an intramuscular injection of ZoletilVet (1.5 mg/10 kg; a mixture of ketamine 8.3 mg/mL, tiletamine 8.3 mg/mL, zolazepam 8.3 mg/mL, butorphanol 1.7 mg/mL, xylazine 8.3 mg/mL) and placed in supine position on a surgical table where they were intubated for ventilation (Dameca Dream). Anesthesia and pain management were then maintained by 6–10 mL/h propofol (10 mg/mL) and 6–10 mL/h fentanyl (50 μg/mL) delivered intravenously by two Alaris GH Plus syringe pumps and combined with a sevoflurane concentration of 1.5% in the inspiration gases (Dräger Vapor 2000). Constant infusion of isotonic saline (0.9% NaCl) was delivered through the same intravenous catheter to prevent dehydration. A Foley catheter with a temperature sensor (Covidien 90055T, Central Infusion Alliance, Inc., Skokie, IL, USA) was inserted into the bladder for urination and monitoring of temperature (T). T was kept constant throughout the experiment by a forced air warming system (Mistral-Air Stryker, Stryker, Portage, MI, USA). After all surgical procedures were completed, sevoflurane anesthesia was reduced to 0.8% and the animal was left to stabilize for 20–30 min before the recordings began. The pigs were monitored throughout the experiment for signs of inappropriate anesthetic level (under supervision by the facility’s responsible veterinarian). If anesthetic management was ineffective and a pig was showing signs of returning consciousness, the experiment would have been terminated and the pig euthanized. No such events occurred during this study. Pigs were fully anesthetized throughout all experimental procedures and euthanized under anesthesia and would therefore only have experienced pain from the pinprick of the initial sedation with ZoletilVet. At the end of the experiment, the animal was euthanized with an overdose of pentobarbital delivered intravenously.

#### 2.1.2. Inclusion and Exclusion of Animals

In total, 15 pigs were used for this exploratory study, which included testing two different types of ECoG electrodes before settling on the MEA electrodes reported in this article. Vital signs were measured during the experiment for ethical purposes (ensuring appropriate sedation) and recorded to validate the primary outcome. Animals presenting systematical drifts in their vitals, in T, would be excluded from further analysis. MEA electrodes were implanted in the primary somatosensory cortex (S1) of eight pigs, in total, where one pig was excluded from analysis because of drift in vital signs.

#### 2.1.3. Preparations for Blood Pressure (BP) and Electrocardiographic Recording (ECG)

An incision was made on the skin along the inguinal ligament to expose the femoral artery, which was freed from surrounding tissues by blunt dissection. Two ligatures (Vicryl, 3/0) were placed around the femoral artery, one at each end of the freed segment. To measure BP and ECG, a (7F, Cordis) catheter was inserted into the artery between the two ligatures using an entry needle (9 cm, 18 G, Cook Medical LLC, Bloomington, IN, USA), and the proximal ligature was then closed around the artery and catheter. The catheter was then sutured to the inguinal ligament to prevent it from sliding out, and the skin was closed. The skin was shaved and cleaned with alcohol before placing ECG electrodes (Ambu BlueSensor, Ambu A/S, Ballerup, Denmark), according to Lead-II.

#### 2.1.4. Peripheral Stimulation Electrode Implant

An incision was made on the right front leg to expose the ulnar nerve, which runs superficially on top of the flexoris digitorum profundi muscle. The ulnar nerve branch was freed from surrounding tissues by blunt dissection, using a noosed glass needle to avoid touching the nerve with conductive materials and being careful not to stretch or crush the nerve. A tripolar cuff electrode (12 mm long, 1 mm wide Pt/Ir ring electrodes embedded in silicone, 5 mm electrode-to-electrode distance, custom-built according to the techniques described by [38]) was placed around the nerve and the opening was sealed with a silicone sheet. Finally, the cuff and seal were secured with three ligatures.

#### 2.1.5. Cortical Electrode Implant

The pig was turned and placed in prone position, and the head was fixed to a stereotaxic frame with screws inserted into the zygomatic bone (the baseplate, mouthpiece and micromanipulator adaptors were designed and 3D printed in our lab, and fitted with Kopf Model 1760 micromanipulator (David Kopf Instruments, Los Angeles, CA, USA) [39]). Incisions were made in the skin along the midline and transversely at the widest point of the scalp. A scalpel was then used to peel the periosteum from the scalp while pulling the skin away from the incisions. The targeted area for craniectomy was then identified, typically extending from 0.5 cm posterior to 3.5 cm anterior of the bregma, and from 0.5 cm right of the midline to 2.5 cm left of the midline. Guide holes were first drilled at each corner of this area using a hand drill (2.5 mm drill bit) and the sides were opened (8228, Dremel, a burr drill bit), using the holes to gauge the thickness of the bone. When nearly through on all sides, a fine-nosed rongeur was used to crack the remaining thin bone and then carefully lift the freed bone, pulling it off the dura. At this point, bleeding from the dura, caused by adhesion to the bone, was stopped by placing absorbable hemostat (Surgicel Fibrillar, Ethicon, Johnson and Johnson MedTech, Warsaw, IN, USA) and bleeding from the skull was cauterized if necessary. A break of about 5 min was taken to wait for the dura bleeding to stop.

Durotomy was performed by first inserting a 23G needle (with a bent tip) into the meninges without touching the cortex. Micro-forceps were then used to hold the dura to cut the dura open. This was performed carefully to avoid cutting vessels under the dura, especially the last few mm near the midline, where the superior cerebral vein must be avoided. The cut-out flap of the dura was folded over the midline and a piece of gauze bandage was soaked in isotonic saline and placed over the dura to keep it moist and retain any remaining blood from the dura.

**Figure 1:**
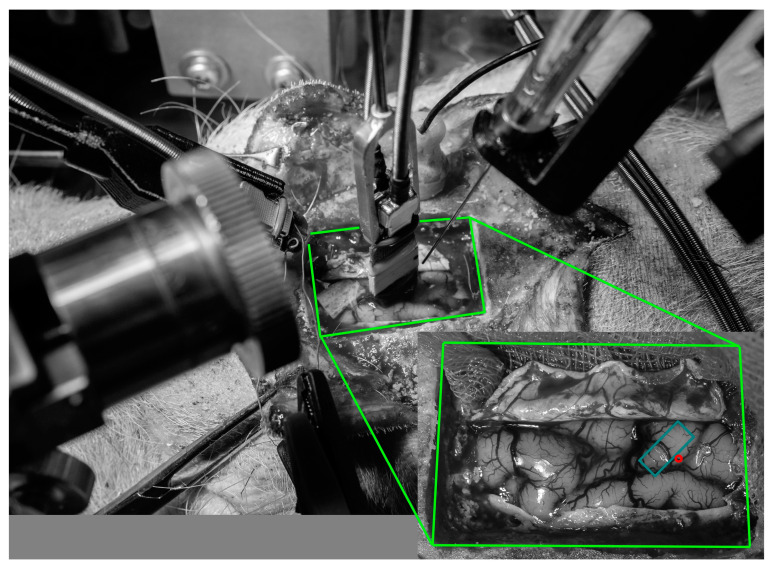
Example of stereotaxic setup following craniectomy and durotomy with the microelectrode array inserted in the forelimb representation of the primary somatosensory cortex (S1) and the Hamilton syringe suspended above the injection site. The cutout shows brain anatomy prior to electrode insertion with the approximate location of the electrode marked by the cyan rectangle (drawn to scale; 3 × 7 mm) and the approximate location of ET-1 injection marked by the red circle. The anterior aspect of the S1 follows the prefrontal gyrus on its lateral and posterior sides and then turns towards the midline.

Micro-manipulators were then secured to the stereotaxic frame. An MEA consisting of 16 (4 × 4, animal 1) or 32 (4 × 8, animal 2–7) Pt/Ir wires (2 mm long, deinsulated tips, 1 mm between each row and column, MicroProbes) was connected to a ZC16 or ZC32 ZIF-Clip headstage (TDT) and suspended in a micro-manipulator. The pig S1 can be morphologically identified as the gyrus between the prefrontal cortex, which follows the midline anterior of S1 all the way to the front of the brain, and the visual cortex posterior of S1 [10] (see Figure 1). The MEA was moved into position above the S1 and lowered until the electrode tips touched the cortex. Then, the MEA was lowered 2 mm and left to settle in the brain for about 30 min before recording. The Hamilton syringe was moved into a position where the tip of the needle could be lowered onto the S1 gyrus, either on the lateral, posterior, or anterior side of the MEA (depending on anatomy and MEA placement) and left with the tip suspended around 2 cm above the brain (see Figure 1).

#### 2.1.6. Intervention with Endothelin-1

As preparation for the intervention, 100 μg of ET-1 (E7764, Merck Life Science, Singapore) was dissolved in 200 μL of isotonic saline (0.5 μg/μL) and then used to fill a 25 μL Hamilton syringe with a blunt needle (Microsyringes). The Hamilton syringe was placed in another micro-manipulator, using a custom-made 3D printed holder.

### 2.2. Stimulation and Recording

Electrical stimulation was delivered through the ulnar nerve cuff electrode every 5 s from the beginning of baseline phase to just before euthanasia. A biphasic stimulus was used consisting of a 2000 μA, 100 μs, cathodic phase followed after 100 μs by a 100 μA, 2000 μs anodic pulse to secure charge balancing (STG4008-16mA MultiChannel Systems, Reutlingen, Germany).

Cortical signals were recorded continuously throughout the experiment. The ZIF-Clip headstage was connected to a PZ5-64 NeuroDigitizer Amplifier (Tucker Davies Technologies, Alachua, FL, USA) where signals were AC-coupled, lowpass-filtered at 10 kHz, amplified, and digitized at 24,414 Hz. Digitized signals were sent via fiber-optic cable to a RZ2-2 Z-Series Bioamp Processor (TDT), where signals were analyzed, and processed data were saved. Raw data were streamed to an RS4 Data Streamer (TDT) for storage. Cortical recordings were visualized and controlled using the Synapse Software Suite (v95, TDT). Cortical signals were synchronized to stimulation by TTL pulses sent from the STG4008 to the RZ2 simultaneously with each cathodic phase.

Heart rate (HR), blood pressure (BP), oxygen saturation (SpO2), T, and end-tidal CO2 (ET) were displayed on a clinical monitor (Datex-Ohmeda S/5) and manually noted every 15 min throughout the experiment, together with respiration rate (RR), sevoflurane concentration (SC), infusion rate of fentanyl (FIR), and propofol (PIR).

### 2.3. Data Analysis

Intracortical signals were filtered from 300 to 5000 Hz (3rd order) and the threshold for spike extraction was set manually within a range of −2.5 to −3.5 standard deviations (SDs). Spikes were detected as threshold crossings with SD calculated over a moving window of 5 s (TDT “auto” mode).

Because the amplitude of neural spikes in our recordings is relatively close to the noise floor, we applied spike clustering as described below as a method for improving the signal-to-noise ratio (SNR) of the signal. This was not intended as a spike sorting and classification step, but only as a way of improving the SNR of the classical Post-Stimulus Time Histogram (PSTH).

**Figure 2:**
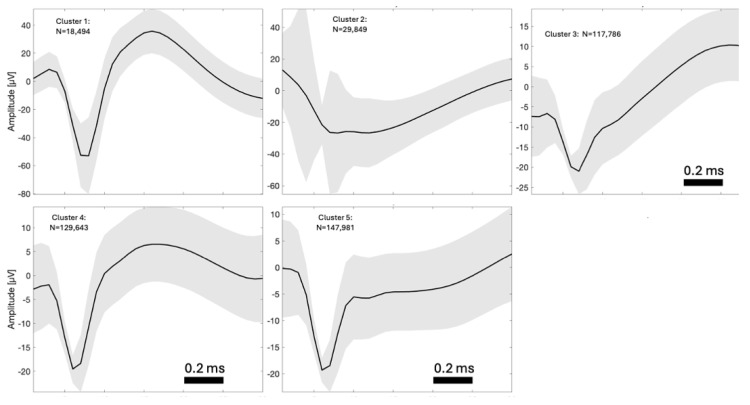
Example of single units identified by the cluster analysis from animal 6, ch. 5 from the baseline period. The black line represents the mean and the grey area represent the individual single units. The number of units (N) is listed for each cluster. Following the single unit identification, the PSTH then was plotted (Figure 3) for each cluster and the cluster was removed from further analysis if it did not show a physiological relevant response. In this case, Cluster 2 and Cluster 3 are not showing clear spike behavior.

Principle Component Analysis (PCA) was performed on the extracted spikes, after which, k-means clustering was applied to the PCA score array with five clusters (see example in Figure 2).

For each animal and cluster, a PSTH then was plotted for the baseline period to manually determine which clusters showed a clear stimulation response and to determine the time window of this response.

**Figure 3:**
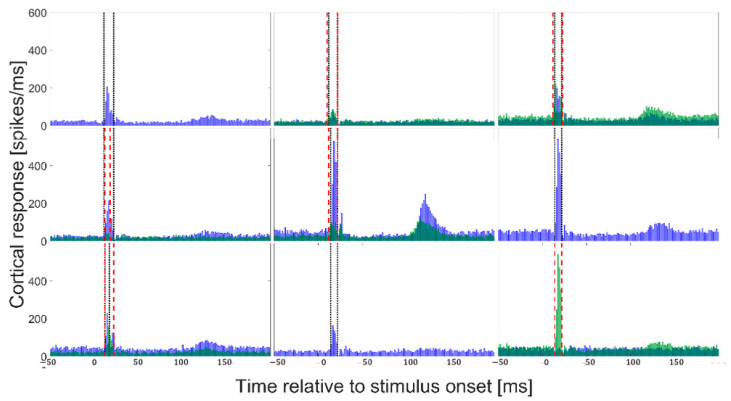
Example PSTH baseline (T0) responses from animal 6, subgroup 9, channel 32 were selected (from the upper right corner of the electrode) for the sake of illustration. PSTHs originating from cluster 1 are plotted in blue, while PSTHs originating from cluster 5 are plotted on top of these in transparent green. When channels were manually selected for further analysis, the dotted lines indicate the start and end of the analysis window (black for cluster 1 and red for cluster 5). Channel 9 did not show a significant response for cluster 1. Channels 1, 6, and 8 did not show a response in cluster 5.

In this way, spikes belonging to clusters that did not display a physiological response as well as spikes not belonging to clusters (i.e., presumed noise spikes) were removed from the analysis (Figure 3). For example, some channels displayed a late response, occurring more than 100 ms after stimulation onset (most notably channel 5, cluster 1, in Figure 3). However, only the first response was included in the analysis of this study and the response window was defined narrowly around this response. Also, some channels would not produce any spikes for clusters 2–5 (as seen for channels 1, 6, and 8 in Figure 3) and were therefore excluded from the analysis. In other cases, a channel cluster was excluded because no significant peak beyond the noise level was seen, as illustrated by channel 9, cluster 1 in Figure 3.

**Figure 4:**
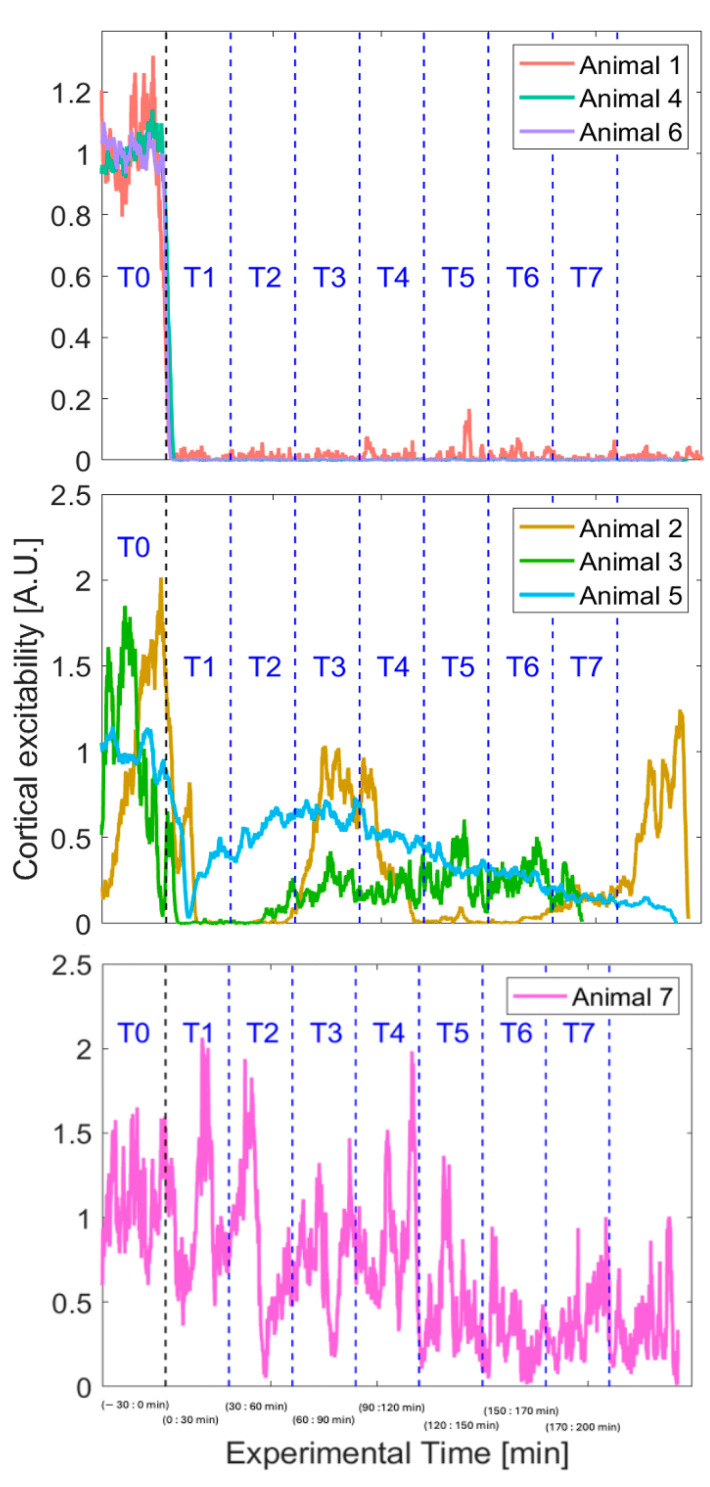
Development in cortical excitability separated into three subgroups of animals. ET-1 intervention took place at 0 min (between T0 and T1). In animals 1, 4, and 6 (top plot), nearly immediate elimination of the cortical response following ET-1 injection was observed. In animals 2, 3, and 5 (middle plot), a strong reduction, but not a permanent elimination, of the cortical response following ET-1 injection was observed. Animal 7 (bottom plot) did not display a clear effect of ET-1 immediately after injection, but the cortical responses did gradually decrease over the following three hours. The vertical stippled black line illustrates the end of baseline, and the vertical stippled blue lines illustrate the boundaries of each 30 min segment, which are labeled in blue text for comparison with Figure 5. The color used for each animal in this figure is identical to the color used for the same animal in the jitter plot in Figure 5.

**Figure 5:**
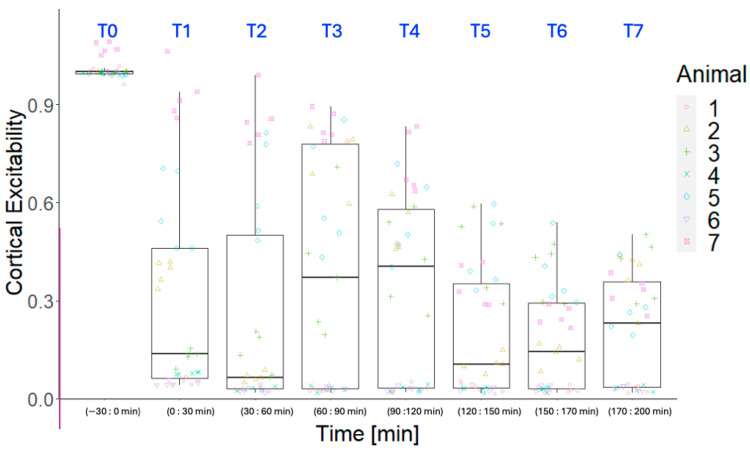
Development in cortical excitability over the experimental timeline, as illustrated by boxes, each representing a 30 min segment of data. The cortical excitability is statistically significantly reduced after baseline (T1–T7).

To extract a global measure of S1 cortical excitability in response to the peripheral stimuli over time, we calculated the normalized firing rate (count of spikes in a 180 s moving window subtracted from the average firing rate of the 50 ms time window preceding stimulation and normalized to baseline). The normalized firing rates were averaged across channels for each pig to represent the global activity, hereafter referred to as “cortical excitability”; see Figure 4 and Figure 5.

The analysis was performed offline in Matlab (R2021a, Mathworks) using the TDT software development kit (v95).

### 2.4. Statistics

To enable testing of the statistical significance of changes related to the time of ET-1 intervention, the experimental data were segmented into bins of 30 min duration from −30 min to +200 min relative to the time of intervention. Hereafter, these bins will be referred to as T0 for baseline (pre-intervention) and T1-T7 for the subsequent 30 min segments after the intervention. ET, HR, mean BP (mBP), SpO2, T, RR, and anesthesia rates were averaged into a single value for each parameter for each 30 min bin if multiple values were available.

To evaluate the data for normality, the Shapiro–Wilk normality test was first employed together with inspection of histograms, box plots, and QQ plots. If data fulfilled normal distribution, the one-way ANOVA was used to test if a significant effect of time exists within each parameter, and if this test was significant, the paired Student’s *t*-test was used for comparing each post-intervention segment to baseline. If data did not fulfill normal distribution, the ANOVA test was replaced by the Kruskal–Wallis rank sum test, and the *t*-test was replaced by the Wilcoxon rank sum test with Bonferroni correction. All statistical tests were performed at the *p* = 0.05 level of significance. Statistical analysis was carried out in R (RStudio Version 1.3.1093).

The Shapiro–Wilk test was not significant for cortical excitability (*p* = 0.212); however, inspection of the histogram and Q-Q plot revealed a tail caused by many values near zero. It was therefore chosen to use non-parametric tests (Kruskal–Wallis and Wilcoxon rank sum test).

The Shapiro–Wilk test showed significance for the vital signs data not following the normal distribution for ET, mBP, HR, RR, SpO2, core body T, SC, FIR, and PIR (*p* < 0.001). It was therefore chosen to use non-parametric tests. It was, however, not possible to perform the Kruskal–Wallis rank sum test on the RR because all the values were identical (15 breaths/min).

## 3. Results

### 3.1. Cortical Response Rapidly Decreased Following ET-1 Intervention

Ulnar nerve stimulation evoked a clear response in 3–30 channels for each cluster of each animal when evaluating the PSTHs of the baseline period (for details, see Table 1). Across all the clusters, 7–32 channels showed responses in each animal. The primary response, which was extracted for this study, lasted 11.1 ± 2.5 ms and occurred 23.0 ± 5.8 ms after stimulation onset.

Three different patterns were observed for the individual animals in the development of cortical excitability following the ET-1 intervention; see Figure 4. In animals 1, 4, and 6, the evoked responses were completely lost within minutes following the ET-1 administration and did not return during the experiment (see Figure 4, top plot). In animals 3 and 5, the cortical excitability was also inhibited by ET-1 administration, but then partially returned, although never to the same level as before the intervention (see Figure 4, middle plot). In Animal 2, we observed a decrease in the average cortical excitability after ET-1 administration; however, the response of this animal appears to be unstable both before and after the intervention. Animal 7 did not display a clear cessation of the evoked responses after ET-1 administration. However, cortical excitability gradually decayed and was clearly below baseline levels from two hours after the intervention (see Figure 4, bottom plot).

The results of the change in the cortical excitability across the animals are illustrated in the boxplot in Figure 5. The significant change in the cortical excitability after ET-1 injection covers a large increase in variability after the intervention compared to baseline. However, the majority of the data points after the intervention are below the mean of the baseline. The Kruskal–Wallis rank sum test showed a significant effect of time on the cortical excitability (*p* < 0.001), and the Wilcoxon rank sum test was therefore used to compare each of the post-intervention segments to the baseline, which showed all the post-intervention segments to be significantly different from the baseline (*p* < 0.001).

**Figure 6:**
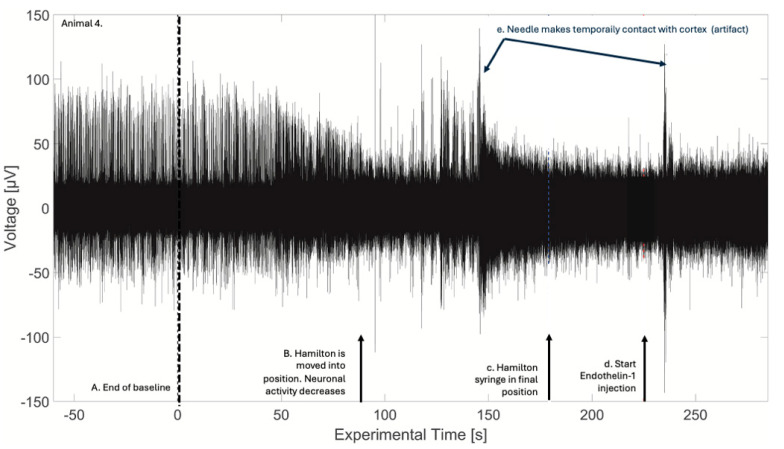
Example of delivery of ET-1. The figure shows the real-time extracellular recording of animal 4, ch. 15, from 60 s before the end of baseline to 60 s after the start of ET-1 injection. Same behavior was seen in all responding channels. A. Time zero and the black stippled line indicate the end of baseline. B. Hamilton is lowered onto the cortex. Since the neuronal activity decreases around 90 s, an ET-1 drop is likely administered inadvertently. C. The Hamilton syringe was in its final position prior to ET-1 injection, and the red stippled line indicates the start of ET-1 injection. The arrows mark what we believe to be movement artifacts caused by the needle briefly contacting the cortex.

The ET-1 application was observed during the experiments and raw signal inspection to affect the cortical signals surprisingly quickly. Figure 6 shows an example from animal 4, where the neuronal spikes seem to disappear from the recording around 90 s after the ‘end of baseline’ (black stippled line) and before the piston of the Hamilton syringe had been pushed (red stippled line). Here, the ‘end of baseline’ denotes the last time that there is known to have been no perturbation of the animal, marked as a note entry in the recording file. After this time point, the lights would be turned on in the surgery room, the experimenter would physically move to the side of the pig, and the tip of the Hamilton syringe needle would be lowered until in contact with the S1 before finally initiating ET-1 injection by pushing the piston of the Hamilton syringe at the time marked by the red stippled line. We observed that a drop would always gather at the end of the needle prior to ET-1 administration. Based on the timing of the loss in the spiking activity seen in Figure 6, it appears that this drop of ET-1 became dissolved in cerebrospinal fluid (CBF) and inhibited neuronal activity promptly upon contact between the needle and CBF, i.e., before pushing the Hamilton syringe piston. We believe that the large spikes seen between 100 and 150 s in animal 6 are artifacts caused by the movement of, e.g., the Hamilton syringe, and the large spikes after the red stippled line (around 235 s) are an artifact from removing the Hamilton syringe after completing ET-1 application. Due to this observation, we chose to use the ”end of baseline” timestamp as time zero for the analysis.

### 3.2. ET-1 Administration Did Not Affect Physiological Parameters

**Figure 7:**
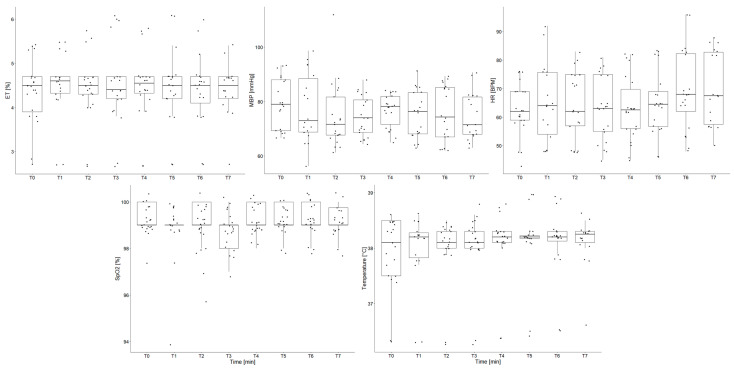
Development in physiological parameters over the experimental timeline, as illustrated by boxes each representing a 30 min segment of data for all animals. ET-1 intervention took place at 0 min (between T0 and T1). The physiological parameters remain relatively stable over the experimental timeline. Anesthesia and RR are not included in the figure since they were set by the experimenter and thus did not fluctuate.

The development over time in the mean value and distribution of the ET, mBP, HR, SpO2, and T is illustrated by the box plots in Figure 7, where each box represents 30 min in the experimental timeline. The variations in the mean value of the ET, mBP, HR, SpO2, and T across the experiment are low compared to the variability within each 30-min segment. The Kruskal–Wallis rank sum test did not reveal any significant differences as an effect of the experimental time for the ET (*p* = 0.997), mBP (*p* = 0.573), HR (*p* = 0.474), SpO2 (*p* = 0.887), T (*p* = 0.970), FIR, and PIR (*p* = 1) or SC (*p* = 0.138).

## 4. Discussion

### 4.1. Cortical Response Rapidly Decreased Following ET-1 Application

The application of ET-1 to the cortical surface caused a reduction in the cortical excitability in all the animals, as expected. In three animals, the cortical responses disappeared almost immediately when the Hamilton syringe came into contact with the CBF. It is worth noticing that the calculated cortical excitability measure represents the average activity across all 16 or 32 channels of the MEA. The abrupt drop therefore indicates that activation was simultaneously blocked within the cortical area covered by the MEA (≥12–21 mm^2^). In the other animals, we found that the drop in the cortical excitability occurred more gradually (Figure 4). We believe that the CBF caused the ET-1 to be distributed beyond the intended area of application. This must also have caused ET-1 to become diluted in an uncontrolled manner, which may explain the inconsistent effect. The variability seen in this manuscript is, however, consistent with that in Wright et al., where ET-1 in only six out of the eleven included pigs decreased the cortical blood flow (CBF) enough to be consistent with the development of an infarct [18].

While ET-1 is cleared quickly from plasma, the main mechanism for this clearance is through binding to ETB receptors [40]. We have not been able to find clear evidence for the clearance time of these bindings. One study in rats indicated that the vasoconstrictive effect of ET-1 may persist for at least 24 h after injection [41]. However, two previous studies in pigs have reported that the effect of ET-1 on CBF may subside within two hours [17,18].

It is unclear from the presented data if reperfusion occurred in this study. Reperfusion may explain the partial recovery of the cortical excitability in animal 3. However, the mechanisms behind the unstable development of excitability in animals 2 and 5 are unclear, and the response in animal 7 indicates a late-onset detrimental effect. The fact that the cortical excitability did not return to the baseline level in animals 1, 4, and 6 does not mean that reperfusion did not occur in these cases. The abrupt cessation in neuronal activation indicates that the ischemic core extended throughout the recording area in these animals.

Some animals displayed a high level of variability in cortical excitability during the baseline period. There are many factors that might contribute to such a change, including the temperature and anesthetic level of the animal. However, our results indicate that physiological parameters were kept stable throughout the experiments.

Since repeatability is vital to the expected application area of our suggested model, we suggest improving the method of ET-1 application in subsequent studies. The issue may be improved by (1) replacing the Hamilton syringe since we found it impossible to avoid droplet formation, (2) applying ET-1 onto a piece of Gelfoam (Pfizer) placed on the cortex to serve as a controlled gateway for transmitting ET-1 to the underlying cortical tissue, (3) injecting ET-1 below the surface of the cortex to obtain a more controlled distribution [22,23,24], or (4) using a micro-infusion pump (example from pigs in [16]).

Ketamine has been shown to improve neuronal survival and to reduce neuronal discharge, see, e.g., [42]. In a systematic review by Telles et al., they compare the ketamine dosages applied to achieve a neuroprotective effect [43]. The review reports infusion rates in swine ranging between 2 and 4 mg/kg/h, and bolus injections between 6 and 200 mg/kg in rats and 30 and 100 mg/kg in mice (and the timing for administration also varied), but it was reported that the dosage needs to be at the high end to have an inhibiting effect on spreading depolarizations. In the present work, ketamine was administered once (1.245 mg/kg) as a premedication at the sub-anesthetic level to achieve sedation, and with the aim to minimize the stress and anxiety of the animals before intubation and shift to inhalation anesthesia. The experimental procedures were initiated no later than 6 a.m. in the morning, and the experimental recordings did not start sooner than 12 p.m., i.e., a minimum of 6 h after the ketamine injection. The half-life for ketamine varies between 1 and 4 h, depending on the species. The ketamine dosage given in the initial sedation was approximately ten times below the recommended dosage for maintaining anesthesia (see, e.g., [44,45]) and below the rates that have been shown to have an inhibiting effect on spreading depolarizations [43,46]. We therefore judge that the ketamine dosage applied in the present work was so low that it had no inhibiting effect on the cortical signals.

### 4.2. Use of Electrophysiology to Assess the Evolution of an Ischemic Infarct

The use of electrophysiological measurements to evaluate the evolution of an ischemic infarct has been applied in other animal models, see, e.g., [47,48,49,50,51]. It is, however, a relatively unusual methodological approach, compared to more traditional assessment measurements (e.g., imaging or histology). Table 2 shows an overview of previous work known to the authors that has applied electrophysiology to monitor the progression of an ischemic infarct in pigs (studies with a focus on hemorrhagic stroke have not been analyzed and not included).

There are three main methodological differences between the previous works and the present study:

(1) All other known studies have utilized MCAO as an infarction method [52,53,54,55,56], whereas we have applied ET-1;
(2) All other studies have utilized ECOG electrodes placed on the dura or cortex to monitor the whole brain, whereas we have used an intracortical array placed in the primary somatosensory cortex (S1) to study a more localized effect;
(3) A high sample rate of 24 kHz was used in the present work, compared to sample ranges between 200 and 2000 Hz, which enabled us to analyze low-frequency spreading depolarizations, local field potentials (or evoked potentials), and single unit activity.

**Table 2:** Comparison of known studies that have applied electrophysiological measurements to monitor the progression of an ischemic infarct in pigs.

First Author (yr)	Title	Animal (Strain)	Intervention/Control or Sham/Used for Model Development	Male/Female	Weight (Age)	Infarction Method	Time	Imaging	Histology or Histopatology	Electrophysiology	Behavioral	Other Data to Monitor Brain Function	Time Points Analyzed	Min Time Resolution of Measurements	Cortical Electrode Type	Cortical Recording Specc	Sample Rate
Kentar (2020) [53]	Detection of speading deploarizations in middle cerebral artery occlusion model in swine	Pig (German landrace)	15/0/7	0/22	32.8 ± 1.47 kg (3–4 mo)	MCA occlusion	acute, 30 h	Intrinsic optical imaging	Infarct volume	ECOG for assessment of spreading depolarizations	no	no	Continuous measurement	Continuous measurements	ECOG	2 × 5 ch platinum strips (Ad-tech, USA), on dura, a strip on each hemisphere	200 Hz
Frasch (2021) [56]	Multimodal pathophysiological dataset of gradual cerebral ischemia in a cohort of juvenile pigs	Pig (mixed breed)	11/0/0	0/11	14.9 ± 1.2 kg (7 wks)	MCA occlusion	unkown	no	no	EEG, EThG Auditory, SEP, high-frequency oscillations	no		Selected events analyzed	Continuous measurement	ECOG	9 channels, Unipolar silver/silver chloride electrodes, covering the whole brain	2 kHz
Frasch (2021) [55]	Update to the dataset of cerebral ischemia in juvenile pigs with evoked potentials	Pig (mixed breed)	11/0/0	0/11	14.9 ± 1.2 kg (7 wks)	MCA occlusion	unkown	no	no	EEG, EThG, SEP	no	Intracrainal pressure	Selected events analyzed	Continuous measurement	ECOG	9 channels, Unipolar silver/silver chloride electrodes, covering the whole brain	2 kHz
Kentar (2022) [54]	Spatial and temporal frequency band changes during infarct induction, infarct progression, and spreading depolarizations in the gyrencephalic brain	Pig (Landrace)	6/0/4	0/10	28–32 kg (3–4 mo)	MCA occlusion	acute, 24 h	no	no	ECOG for assessment of spreading depolarizations + activity in delta, theta, alpha, beta and gammaband	no	no	5 min, 4 h, 8 h, 12 h	Continuous measurements	ECOG	2 × 5 ch platinum strips (Ad-tech, USA), on dura, a strip on each hemisphere	>200 Hz
Sanchez-Porras (2022) [52]	Eighteen-hour inhibitory effect of s-ketamine on potassium- and ischemia-induced spreading depolarizations in the gyencephalic swine brain	Pig (German landrace)	16/11/0	0/27	30–35 kg (3–4 mo)	MCA occlusion or KCl stimulation	acute, up to 21 h	Intrinsic optical imaging	no	ECOG for assessment of spreading depolarizations	no	Laser speckle flowmetry (CBF)	Selected events analyzed	Unknown	ECOG	2 × 5 ch platinum strips (Ad-tech, USA)	200 Hz
Present work, Nielsen (2024)	Porcine model of cerebral ischemic stroke applying intra-cortical recordings for the continuous monitoring of the ischemic area	Pig (Danish landrace)	7/0/8	0/15	32.7 ± 3.1 kg (12 wks)	ET-1 injection on cortex surface	acute, 3 h	no	no	Use of SEP and Intra-cortical recordings-cortical for analysis of cortical exictability	no	no	Baseline, continuous measurements of cortical signals, physiological variables approx every 30 min	Continuous measurements	MEA	16 or 32 ch Pt/Ir wires (Microprobes) inserted in S1	24 kHz

Since we are recording from a localized brain area, we can only investigate the localized effects, but we believe that this would be an advantage in studying neuroprotective agents. Neuroprotection in ischemic stroke covers methods that reduce detrimental processes in the brain through means other than re-establishing cerebral blood flow [4]. Over the past decades, multiple pharmaceuticals have been identified and have shown great promise in (mostly rodent) animal models, but their successful implementation in clinical trials has remained elusive [4,7,8]. While each neuroprotective agent acts in a specific way, their effectiveness relies on inhibiting aspects of the ischemic cascade with anti-inflammation, antioxidation, vasodilation, and inhibition of excitotoxicity being among the main mechanisms of neuroprotection, and most agents act on multiple of these mechanisms [4]. The timing issue of the intervention may be important for neuroprotective agents that mitigate the effects of, e.g., excitotoxicity [6]. Furthermore, the potential for salvageable tissues is far greater within the first hour [5]. We believe that the methodology that we present here would be able to ‘catch’ fast changes in the progression—or inhibition—of cortical ischemic stroke and thereby serve as an evaluation tool.

### 4.3. Future Implications

Our results should be validated in (1) a more physiologically relevant stroke model (e.g., ET-1-based MCA model) with relevant comorbidities, and (2) a chronic adaptation of the model, in which a histological endpoint can be obtained before transitioning into human applications. The pig model enables the multi-dimensional recording and analysis of clinically relevant measurements such as blood gas testing and lead-12 electrocardiographic (ECG) recording [55,56], cerebral blood flow measurements (e.g., laser speckle contrast imaging [57]), or cerebral micro dialysis [58]. It could also be interesting to expand upon the model presented here by recording multiple centers of the brain (e.g., the motor cortex, secondary sensory cortex, anterior cingulate cortex, and hypothalamus) ipsilaterally or even in the contralesional hemisphere [59] to investigate the disruptive effects of stroke on connected areas with unaffected perfusion.

## 5. Conclusions

We believe that intracortical recordings for the continuous monitoring of an ischemic infarct provide a route for monitoring cortical neurons in ischemic and penumbral tissues with a high resolution in the time domain. The variability seen after ET-1 application in this study may indeed indicate the method’s ability to show differences in infarct development early. With appropriate improvements, the model may provide an interesting platform for screening the early neuroprotective effects of candidate agents in an animal model of relative resemblance to human neurological and cardiovascular physiology as well as body size, thus easing the translation of results to human applications.

## Figures and Tables

**Table 1:** Evaluation of PSTH properties in the baseline period (T0). The number of responsive channels is given for each cluster (C1–C5), as well as across clusters, and response timing is presented as the mean delay and width of the response window (manually defined by inspection of each PSTH) for each animal. The number of ‘unique channels’ is defined as the number of channels that shows a response in at least one cluster.

Pig	Number of Included Channels						Response Window [ms]	
	C1	C2	C3	C4	C5	Unique	Mean	Width
1	9	5	3	6	6	11	24.5 ± 3.3	13.8 ± 4.2
2	27	10	11	12	12	32	28.1 ± 1.1	9.5 ± 2.6
3	3	4	6	4	4	7	32.8 ± 2.6	15.0 ± 4.5
4	25	25	25	23	30	32	18.5 ± 1.2	8.8 ± 3.0
5	20	16	14	12	11	31	18.0 ± 3.3	9.7 ± 5.1
6	24	25	21	25	23	31	17.4 ± 2.0	9.2 ± 2.9
7	14	11	11	8	11	20	21.5 ± 2.1	11.7 ± 4.1
Mean	17.4	13.7	13.0	12.9	13.9	23.4	23.0 ± 5.8	11.1 ± 2.5

## Data Availability

The data presented in this study are available on request from the corresponding author.
